# Sacrococcygeal chordoma with spontaneous regression due to a large hemorrhagic component

**DOI:** 10.1007/s00256-024-04700-9

**Published:** 2024-05-13

**Authors:** Tania Marlem Chico González, Suk Wai Lam, Robert van der Wal, Ana Navas, Kirsten van Langevelde

**Affiliations:** 1 LUMC: Leiden University Medical Center, Leiden, Netherlands; 2 Department of Radiology, Hospital Universitario de Canarias, Santa Cruz de Tenerife, Spain; 3 Department of Pathology, Leiden University Medical Center, Leiden, Netherlands; 4 Department of Orthopaedic Surgery, Leiden University Medical Center, Leiden, Netherlands; 5 Department of Radiology, Leiden University Medical Center, Leiden, Netherlands

**Keywords:** Chordoma, Bone tumor, Bleeding, Hemorrhagic, Spontaneous regression, Magnetic resonance imaging

## Abstract

Chordoma is a malignant bone tumor originating from notochordal remnants, most commonly occurring at the sacrococcygeal junction. We present a case of a 70-year-old male with chronic pain in the lower lumbar spine. MRI performed elsewhere revealed a large tumor that involved S4, S5, and the coccyx with a presacral soft tissue component. The lesion was heterogeneously hyperintense on T2-weighted images with a thick hypointense rim anteriorly. On T1-weighted images, the lesion showed a native hyperintense signal centrally probably due to hemorrhage. Based on this MRI, the diagnosis of chordoma was suggested. A spontaneous marked reduction in size was observed on a 4-week interval MRI performed at our institution before biopsy. Due to spontaneous tumor shrinkage along with peripheral enhancement, a differential diagnosis of infection or bleeding in a retrorectal cyst was proposed. This case teaches us that chordomas may contain a large hemorrhagic component, which is hyperintense on T1-weighted images and shows peripheral rim enhancement. Spontaneous shrinkage of a tumor may occur due to the resolution of a hematoma within weeks. Biopsy is key to obtain the correct diagnosis. Understanding the typical and more rare features of chordomas is key for MSK radiologists as well as pathologists. Chordomas are typically slow-growing tumors, but radiologists should be aware that intratumoral hemorrhage can lead to rapid changes in tumor size, which may be mistaken for either regression or progression of tumor. This case highlights the importance of considering hemorrhagic events within chordomas in the differential diagnosis when observing size fluctuations on imaging.

## Introduction

Chordomas typically present as midline or paramedian slow-growing malignant tumors, predominantly in the sacrococcygeal region and the skull base, less frequently in the mobile spine and craniocervical junction. These tumors are characterized as tumors with a local osseous destructive behavior. The tumor mostly occurs in patients over 50 years of age. The clinical presentation of a sacrococcygeal chordoma includes pain, numbness, and bowel or bladder dysfunction, correlating with tumor location and size. On conventional radiographs or CT imaging, a chordoma typically manifests as a destructive osteolytic sacrococcygeal mass with commonly secondary soft tissue extension. Internal calcifications may be observed in the tumor, but these are due to the presence of “pre-existing trapped bone” as these tumors are not matrix-forming tumors. On MRI, the combination of a tumor with a high signal intensity on T2-weighted images and a multilobulated sacrococcygeal mass is strongly suggestive of a chordoma. Areas of intrinsic hyperintensity on T1-weighted images in chordomas typically represent areas of hemorrhage or mucinous material [1]. This case introduces an unusual course of disease progression, providing insights into the variable natural history of chordomas, particularly regarding size fluctuation and its implications on management strategies. Differential diagnoses will be discussed.

## Case report

**Fig. 1:**
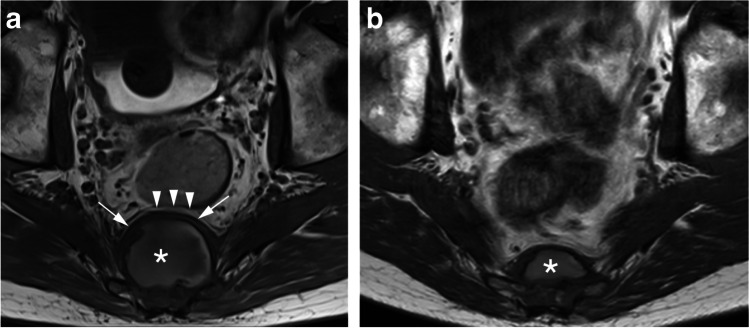
Baseline (**a**) and follow-up (**b**) axial non-contrast T1-weighted images. **a** Presacral soft tissue component with a native hyperintense signal centrally (*) and a thick hypointense rim on the anterior side (white arrows). The lesion causes a mild mass effect over the rectum with a preserved fat plane in between the tumor and the rectum (arrowheads). **b** Note the marked decrease in size occurring in 4 weeks time. The signal intensity in the soft tissue mass has not changed (*)

**Fig. 2:**
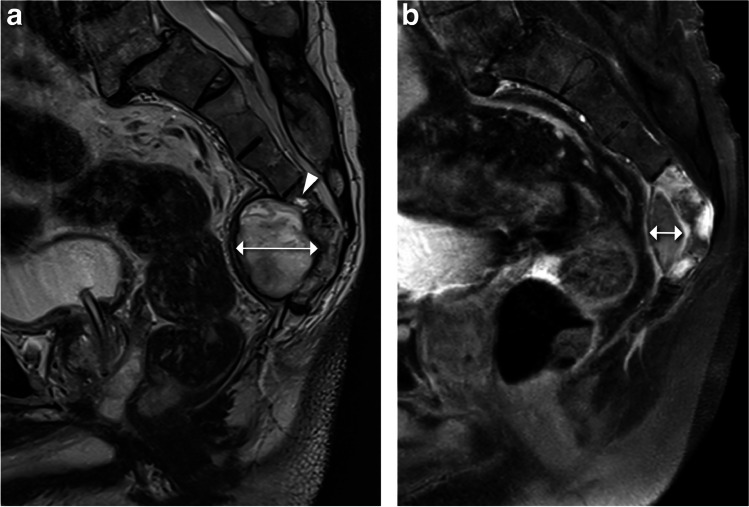
Decrease in size of the tumor’s presacral soft tissue component. **a** Baseline sagittal T2-weighted MR image shows a focus of hyperintense signal intensity within the marrow of S4 (arrowhead). This finding, alongside the anterior soft-tissue component of the mass, points towards the osseous origin of the tumor. On the baseline MRI, the anteroposterior diameter of the soft tissue mass is 47 mm (double arrow). **b** Follow-up sagittal contrast-enhanced T1-weighted fat-saturated MR image shows a decrease in size of the anterior soft tissue component (anteroposterior diameter is 16 mm) with rim enhancement and heterogeneous enhancement of intraosseous component

A 70-year-old man presented with chronic lower back pain. He had no relevant medical history and did not receive any medication. The patient experienced no fever, and his blood tests were unremarkable, especially regarding infection/inflammation. MRI revealed a large lesion that involved S4 and S5 and the coccyx with a presacral soft tissue component. On T1-weighted images, the lesion showed a native hyperintense signal centrally, which may be due to previous hemorrhage (Fig. 1a). The sacral roots S1–S3 appeared to be free of tumor on both sides but roots S4 and caudally could not be demarcated from the tumor. The lesion had mass effect over the rectum without invading it (Fig. 1a). The lesion was heterogeneously hyperintense on T2-weighted images with a thick hypointense rim around the presacral soft tissue mass (Fig. 2a). After contrast predominantly peripheral, heterogeneous enhancement of the soft tissue mass was seen with diffuse and more solid enhancement of the intraosseous component of the tumor (not shown)*.*

**Fig. 3:**
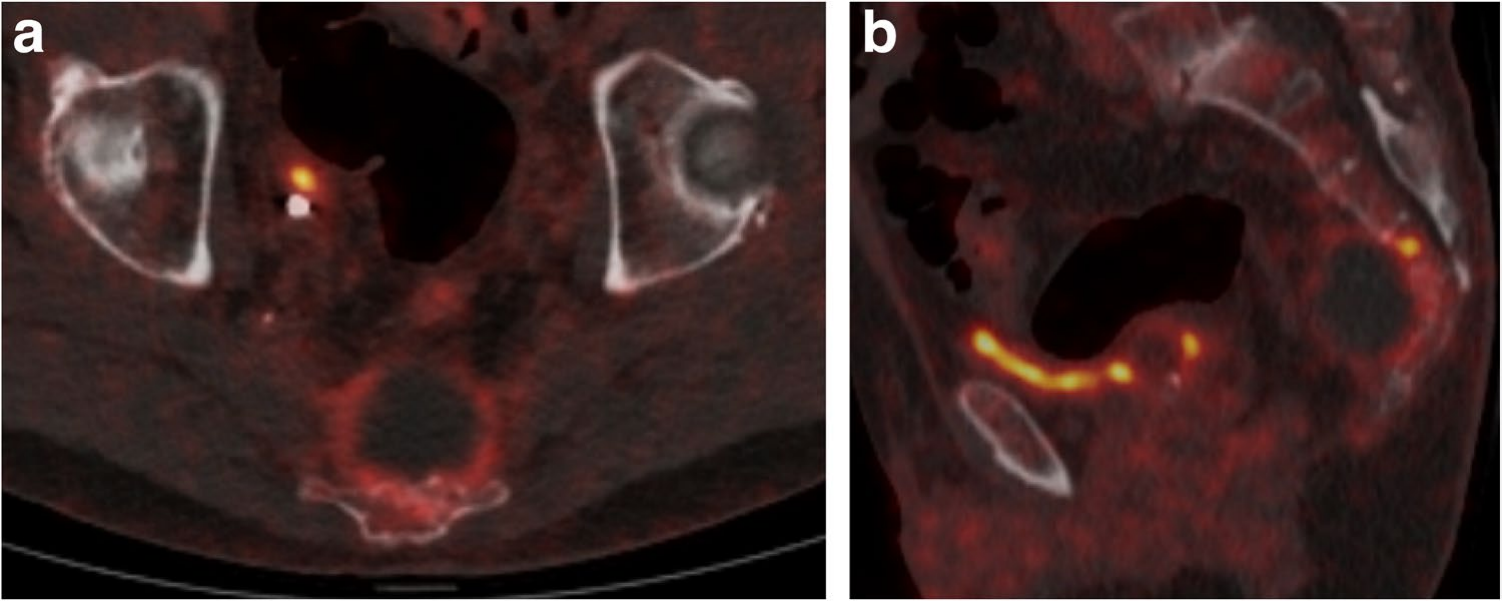
Fused ^18^F-FDG PET-CT images of the sacrococcygeal tumor at baseline. **a** Axial image shows a peripheral rim of FDG uptake in the soft tissue mass. **b** Sagittal image shows an osteolytic tumor with moderately increased metabolic activity in the rim of the soft tissue component. There is discrete cortical destruction at level S4 with a focus of high FDG uptake within the marrow

On the ^18^F-FDG PET-CT, the lesion showed a predominantly osteolytic aspect. No matrix mineralization was demonstrated in the presacral soft tissue component*.* There was mild FDG uptake peripherally in the tumor (Fig. 3 a and b).

Due to the patient’s age, tumor location, and characteristics on PET-CT and MRI, the radiological diagnosis of chordoma was made. However, as the initial study came from an external hospital, a dedicated MRI examination of the sacrum including diffusion-weighted and dynamic post-contrast sequences was performed at our tertiary sarcoma center. The time interval between the two MRIs was approximately 4 weeks, and during this time, the patient had not received any treatment.

**Fig. 4:**
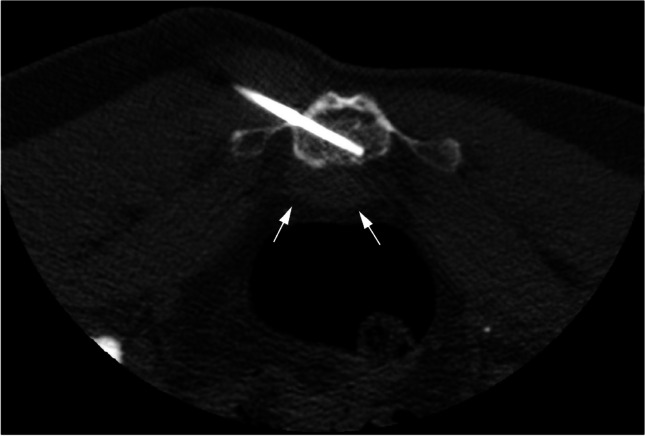
Axial image of the CT-guided biopsy (with the patient in a prone position). The biopsy was taken from the bone (see the needle tip) to prevent a false negative biopsy result from the presacral soft tissue mass which had spontaneously decreased in size (arrows)

A notable spontaneous decrease in tumor size was identified on the subsequent MRI (from 46 × 47 × 45 mm to 37 × 16 × 33 mm) (Figs. 1b and 2b). This gave rise to a new differential diagnosis including an infectious or inflammatory process, such as osteomyelitis with a presacral abscess or a presacral cystic process with reactive changes in the adjacent bone, such as a presacral epidermoid cyst or retrorectal cystic hamartoma. Finally, a CT-guided biopsy was performed. The biopsy was taken from the bone (see the needle tip) to prevent a false negative biopsy result from the presacral soft tissue mass which had spontaneously decreased in size (Fig. 4).

Hematoxylin and eosin (H&E) stain showed large tumor cells with a distinct cellular membrane arranged between pre-existing lamellar bone. The tumor cells contained abundant eosinophilic cytoplasm with intracytoplasmic vacuoles. Nuclei were round to round oval and showed slight variation in size. Additional brachyury staining showed strong nuclear staining, confirming the diagnosis of a conventional chordoma.

**Fig. 5:**
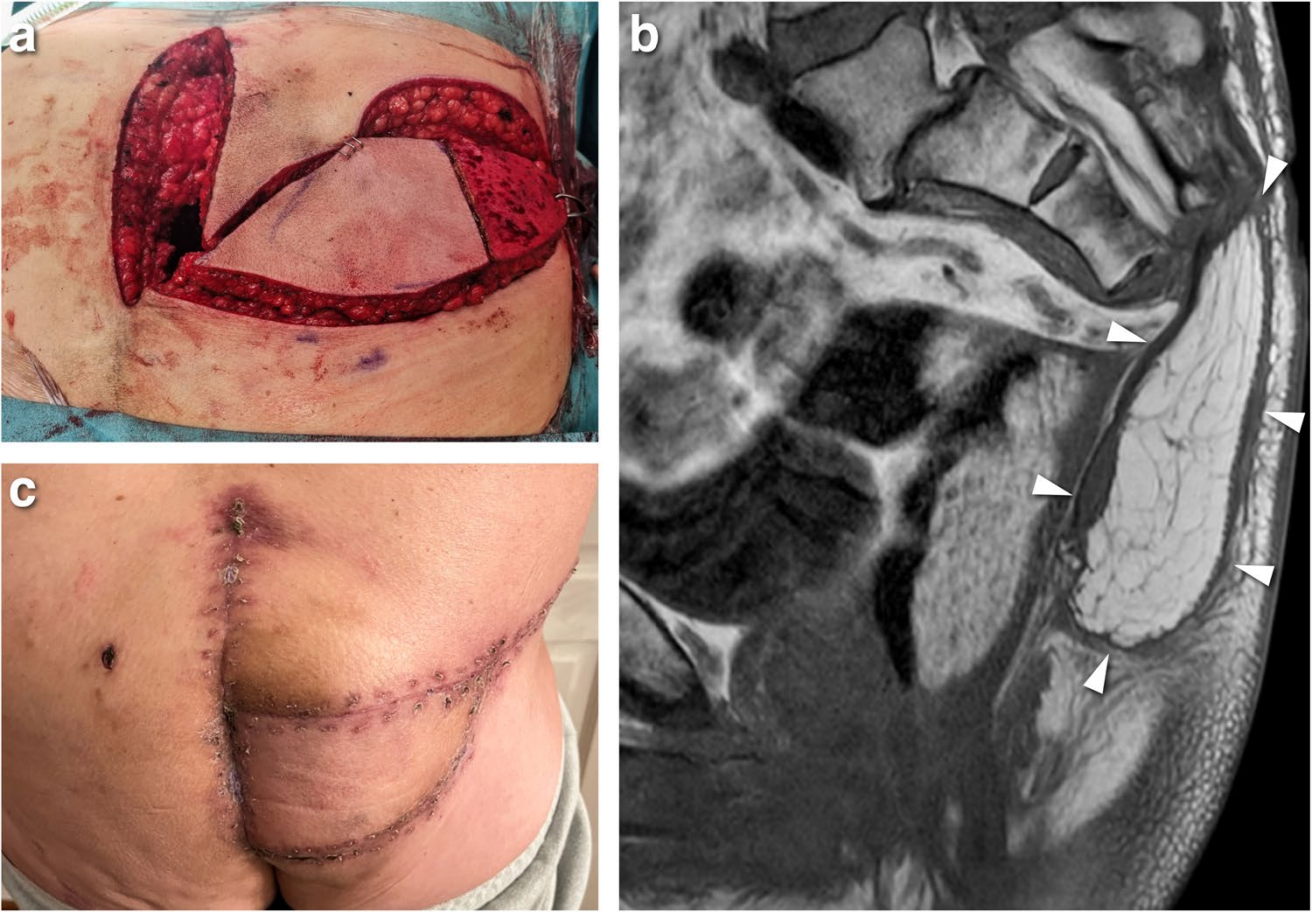
Preoperative and postoperative images. **a** Preoperative photograph during the soft tissue reconstruction using a partially de-epithelialized superior gluteal artery perforator (SGAP) flap. **b** Postoperative sagittal non-contrast T1-weighted image shows sacrectomy below the S2 level and soft tissue reconstruction using the SGAP flap (arrowheads). **c** Clinical image 6 weeks postoperatively showing uneventful healing of the SGAP flap

The patient was treated with neoadjuvant proton beam radiotherapy and a computer-assisted partial sacrectomy below the S2 level, saving S3 nerve roots bilaterally. A soft tissue reconstruction was performed using a partially de-epithelialized superior gluteal artery perforator (SGAP) flap to fill the void and provide primary wound healing (Fig. 5a). Wound healing was uneventful, and postoperative MRI showed no complications regarding the flap (Fig. 5 b and c).

**Fig. 6:**
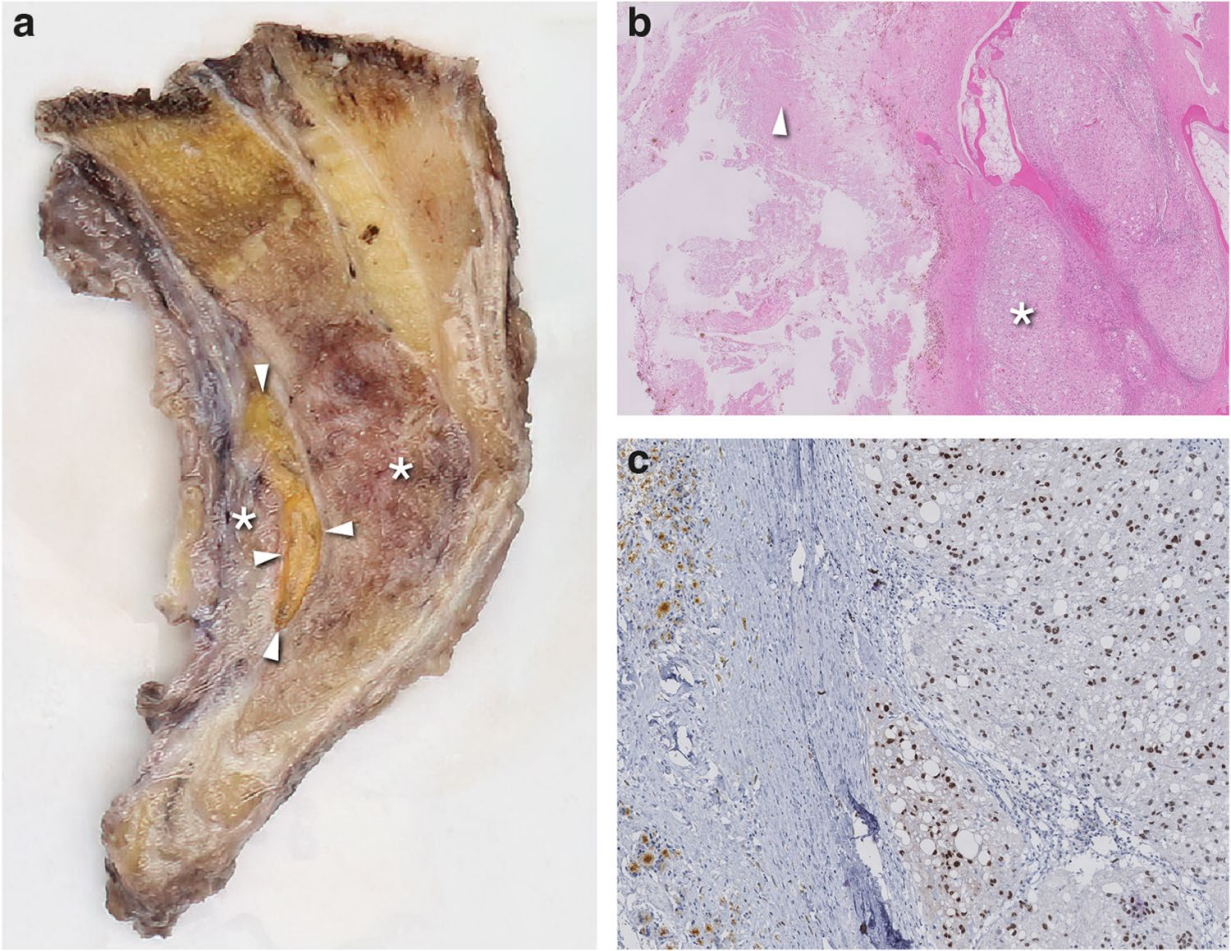
Pathology images after sacrectomy. **a** Sagittal section of the sacrectomy specimen shows a poorly demarcated glassy tumor in the sacral bone and in the soft tissue (*). A presacral area with hemorrhage is noticeable (arrowheads) and corresponds to the MR image of Fig. 2b. **b** On the H&E section, this hemorrhagic area is composed of fibrine and iron-laden macrophages (arrowhead). Tumoral cells adjacent to this area showed abundant eosinophilic cytoplasm with intracytoplasmic vacuoles with nuclei varying in shape and size (*). **c** Brachyury staining was positive in the tumoral cells, confirming the diagnosis of chordoma

The sagittal section of the sacrectomy specimen shows an ill-defined glassy tumor in the sacral bone, as well as in the soft tissue. Between the tumor process, a sharply defined area is noticed with a striking yellow color, suspicious for post-hemorrhagic changes (Fig. 6a). On the H&E section, this area was composed of fibrine and iron-laden macrophages, with the tumor cells adjacent to this area showed similar morphology as was seen on the pre-operative biopsy (Fig. 6b). Additional brachyury staining was positive in the tumoral cells and negative in the area with bleeding (Fig. 6c).

Four months after surgery, the patient reported new complaints of pain in the sacroiliac joint on both sides. A new follow-up MRI showed bilateral insufficiency fractures of the remaining sacrum (images not shown).

## Discussion

Chordomas represent 2–4% of all primary malignant bone tumors and are the most common primary malignant sacral tumor [1]. They arise from remnants of embryonic notochordal tissue. Patients are most frequently diagnosed in their fourth to seventh decade with a peak in the fifth decade [1]. There is a male predilection. Despite their malignancy, chordomas rarely metastasize but are known to be locally aggressive and have a high local recurrence rate [2, 3]. The tumor is slow growing, which accounts for the relatively large tumor sizes seen in the sacrococcygeal region at presentation.

On imaging, a chordoma typically manifests as a large destructive sacrococcygeal mass with secondary soft tissue extension. Radiographs may show osteolysis with an associated soft-tissue mass and sparse calcifications, consistent with “pre-existing trapped bone.” However, these tumors may easily be missed on conventional imaging due to overprojection of bowel gas. On CT, bone destruction with an associated lobulated midline soft-tissue mass is typically seen. CT is useful for defining the extent of bone involvement. Chordomas tend to show hypointense or isointense signal relative to muscle on T1-weighted images. Areas of intrinsic T1 signal hyperintensity typically represent hemorrhage or mucinous material [1]. On T2-weighted images, chordomas show high signal intensity (high water content). After contrast, mild heterogeneous and often peripheral enhancement of the tumor are present.

The phenomenon of spontaneous regression (SR) in chordomas is exceedingly rare, with only a few reported cases in literature (to date, cases of SR of clival chordomas [2, 3], cervical chordomas [4], and pulmonary metastases in a sacral chordoma [5] have been published). SR is defined as the partial or complete disappearance of a malignant tumor in the absence of therapy or with inadequate treatment [5]. Our patient did not undergo any medicamentous treatment during the time period wherein the SR occurred.

The mechanism behind SR in malignant tumors is unclear, and several possible causes have been reported, including immunological or endocrine diseases [2, 4, 5]. The present case was interpreted by us as a spontaneous partial regression due to a decrease in the hemorrhagic component. The decrease in size of the hemorrhage occurred as fast as in 4 weeks. The etiology of chordoma hemorrhage is poorly understood but has been reported in the literature, mainly in clival chordomas [3, 6]. Some authors have reported that rapid tumor growth without adequate matched blood supply may cause small friable vessels to rupture, and vascular proliferation and subsequent occlusion of smaller vessels may also cause tumor necrosis and hemorrhage [6]. To our knowledge, this is the first case of sacrococcygeal chordoma with SR due to a reduction in its hemorrhagic component described. Of note, chordomas may contain variable amounts of hemorrhagic components; we showed an example of intratumoral hemorrhage resorption over a limited time period. However, this does not imply that the tumor itself went into regression.

In conclusion, this case report highlights an atypical manifestation of sacrococcygeal chordoma, characterized by SR in tumor size due to partial resolution of an area of hemorrhage. This case challenges the conventional understanding that chordomas are slow-growing tumors. It demonstrates that they can undergo rapid size changes, potentially leading to misinterpretation in clinical and radiological assessment. Therefore, it is crucial for a MSK radiologist to consider a hemorrhagic component in the differential diagnosis of sacral masses with variable dimensions of the soft tissue components.
